# Demographics-robust spontaneous eye blinking slowing in patients with severe acquired brain injury

**DOI:** 10.1093/nc/niag043

**Published:** 2026-08-12

**Authors:** Alfonso Magliacano, Leonardo Corsi, Piergiuseppe Liuzzi, Calogero Maria Oddo, Andrea Mannini, Anna Estraneo

**Affiliations:** IRCCS Fondazione Don Carlo Gnocchi ETS, Via di Scandicci 269, 50143 Firenze, FI, Italy; IRCCS Fondazione Don Carlo Gnocchi ETS, Via di Scandicci 269, 50143 Firenze, FI, Italy; The BioRobotics Institute, Scuola Superiore Sant’Anna, Viale Rinaldo Piaggio, 34, 56025 Pontedera PI, Italy; IRCCS Fondazione Don Carlo Gnocchi ETS, Via di Scandicci 269, 50143 Firenze, FI, Italy; The BioRobotics Institute, Scuola Superiore Sant’Anna, Viale Rinaldo Piaggio, 34, 56025 Pontedera PI, Italy; IRCCS Fondazione Don Carlo Gnocchi ETS, Via di Scandicci 269, 50143 Firenze, FI, Italy; IRCCS Fondazione Don Carlo Gnocchi ETS, Via di Scandicci 269, 50143 Firenze, FI, Italy

**Keywords:** severe acquired brain injury, disorders of consciousness, eye blink, electro-oculography, rehabilitation

## Abstract

This study aimed to explore whether spontaneous eye blinking features from a brief vertical electro-oculogram recording can distinguish patients with severe acquired brain injury from healthy volunteers. Given evidence for shared neurobiological substrates in blinking control and attention, we expected altered blinking features in patients. In this observational cross-sectional study we extracted eye blink rate, amplitude, rise time, fall time, duration, and blink-blink interval variability from electro-oculography in 19 patients with severe acquired brain injury and 21 healthy controls during resting state and an auditory oddball task. We tested group differences with Kruskal-Wallis and Conover-Iman post-hoc comparisons. Differences covarying age and sex were assessed with Wald tests for odds ratios from logistic regression. Univariate Kruskal-Wallis testing showed longer rise time and duration and smaller amplitude in patients relative to healthy controls. When considering demographics as covariate, we show that amplitude is strongly confounded by demographics whereas rise time, duration and, to a lesser extent, fall time, are associated to the brain injury condition regardless of age or sex. Eye blink rate and blink-blink interval variability did not show statistically significant differences. Condition moderated effects: group contrasts were strongest at rest and attenuated during oddball. We found preliminary evidence that rise time and total duration are prolonged in patients independently of demographic factors, although with no difference between different levels of consciousness. Our results suggest a general slowing of blink dynamics consistent with altered basal ganglia dopaminergic activity, contributing physiological information relevant for monitoring and rehabilitation planning in brain injury.

## Introduction

Severe acquired brain injuries (sABI) commonly result in disorders of consciousness, including Vegetative State/Unresponsive Wakefulness Syndrome (*VS*/UWS), i.e. awake but unconscious patient (Laureys *et al*. 2010) and Minimally Conscious State (MCS), i.e. awake patient with minimal but reproducible behavioural sign of consciousness (Giacino *et al*. 2002), which, when lasting at least 28 days post-injury, are termed prolonged disorders of consciousness (pDoC) (Giacino *et al.* 2018). With recovery, patients may emerge from MCS (eMCS) with residual instrumental cognitive deficits, most often in attention, memory, processing speed, or visuospatial functions, as frequently reported after both traumatic (Tsai *et al.* 2021) and non-traumatic (Bodien *et al.* 2020) brain injury. An accurate assessment, by clinical assessment and both qualitative and quantitative EEG (Estraneo *et al.* 2020; Ballanti *et al.* 2022) of cognitive functions, including the level of consciousness in patients with pDoC, is a cornerstone to plan care and rehabilitation pathways and to identify residual cognitive resources that may support recovery (Lavezzi *et al.* 2022).

However, standardized cognitive assessment can be challenging at all stages of recovery from sABI. Indeed, in addition to fluctuations in arousal, the co-existence of severe sensory-motor impairments, may hinder the recognition of conscious behavioural responses in pDoC, and prevent the access to formalized neuropsychological testing to conscious eMCS patients. Specifically, the clinical recognition of awareness and partially preserved cognitive functions can be prone to error, resulting in a phenomenon known as ‘covert awareness’ or ‘cognitive-motor dissociation’, in which signs of aware cognitive activity can only be detected through advanced instrumental methods like functional neuroimaging (Schnakers *et al.* 2022; Bodien *et al.* 2024). Given the limited availability of these techniques and of specialized expertise for their application in clinical practice, there is an urgent need of novel, complementary and easily applicable bedside tools to assess cognitive processes in patients with sABI.

Since the pioneering studies (Ponder and Kennedy 1927), spontaneous eye blinking has gained attention as a potential index of cognitive activity, beyond its classical role in maintaining ocular surface moisture (McMonnies 2010). This behaviour, being largely involuntary and requiring minimal patient cooperation, is an attractive candidate for use in populations where voluntary behavioural responses are limited or absent. In healthy individuals at rest, spontaneous eye blinks occur every 3–5 s, for an average eye blink rate (EBR) of ~20 blinks/minute, notwithstanding a large inter-individual variability (Nakano 2017). During experimental conditions, the EBR has been shown to be sensitive to cognitive load (Magliacano *et al.* 2020) and to attentional focus (Magliacano *et al.* 2023), in healthy individuals. It has been also demonstrated that conscious individuals with acquired brain frontal cortex injury exhibit a significantly higher EBR modulation, expressed as an increased relative difference between EBR collected during a cognitively demanding task (i.e. mental arithmetic) and blinking collected at rest (Anagnostou *et al.* 2011).

Spontaneous eye blinking is related to the activity of the trigemino-spinal complex, modulated by basal ganglia dopaminergic activity *via* the superior colliculus and the nucleus raphe magnus (Kaminer *et al.* 2011). Indeed, indirect evidence suggests a relationship between EBR and dopamine level in the central nervous system (Jongkees and Colzato 2016). For instance, the decrease of dopamine in the nigro-striatal projections observed in Parkinson’s disease is reflected in a reduced EBR (Karson 1983; Karson *et al.* 1984), compared to healthy individuals. Conversely, dopamine excess in meso-cortical pathways, as in individuals with schizophrenia, leads to an increase in EBR (Karson *et al.* 1990; Chan and Chen 2004). According to the mesocircuit hypothesis (Schiff 2010), dopaminergic projections within striato-thalamo-cortical circuits also play a role in regulating conscious processes. These findings have opened the possibility of using spontaneous blinking as a behavioural biomarker of residual consciousness in patients with pDoC. A previous work demonstrated that resting EBR can differentiate patients in UWS from those in MCS, as it is significantly higher in patients in MCS (Magliacano *et al.* 2021).

Studies in healthy individuals have suggested that, beyond EBR, further blink features (e.g. amplitude, duration, and inter-blink variability) are also modulated by attentional load (Casse *et al.* 2007; Kapitaniak *et al.* 2015; Talens-Estarelles *et al.* 2022). Specifically, blink amplitude and temporal course (rise and fall times) may index subtle changes in motor output or central drive associated with task engagement (Casse *et al.* 2007; Talens-Estarelles *et al.* 2022). To our knowledge, no study has yet systematically explored these additional blink features in patients with sABI, nor their possible value to discriminate different levels of consciousness.

The aim of this study was to explore whether a detailed assessment of spontaneous blinking could serve as an objective and clinically informative tool for evaluating quantitative physiological correlates of cognitive activity in patients with sABI.

To this end, we examined specific spontaneous blink features, including EBR, amplitude, duration, rise and fall time, and blink-blink interval variability in a sample of sABI patients and compared them to healthy individuals. Moreover, as previous literature showed an influence of cognitive activity on blinking, we investigated these features during both a rest condition and an active auditory oddball paradigm.

## Methods and materials

This study has an observational, cross-sectional design. The institutional review board reviewed and approved the project. This study was then approved by the ethical standards committee Campania 3 (protocol n°00007507 of the 05/03/2025) and has been registered on ClinicalTrials.gov (NCT06464549). The study has been conducted according to the ethical standards of the Declaration of Helsinki and its later amendments. Written informed consent was obtained from the participants or from their legal representative/primary caregiver when the patients could not give the consent themselves.

### Participants

We enrolled a convenience sample of patients with sABI from an intensive neurorehabilitation unit for sABI at Fondazione Don Carlo Gnocchi ONLUS in Sant’Angelo dei Lombardi (Italy) from 05 March 2025 to 17 July 2025, including both patients with pDoC and eMCS. Inclusion criteria were: i) age ≥ 18 years; ii) time post-injury (TPI) ≥28 days; iii) written informed consent by patient or, where necessary, by legal representative/primary caregiver. Exclusion criteria were: i) large craniectomy interfering with EOG recording; ii) severe clinical complications (e.g*.* status epilepticus); iii) presence of ophthalmic disorders or impairments in eyelid motility on clinical evaluation; iv) administration of sedative medication in the previous 24 h that could impact on cognitive activity and arousal level; v) previous history of neurological or psychiatric diseases.

In parallel, we enrolled a benchmark population of healthy individuals, following a snowball sampling by means of word-of-mouth. This population underwent anamnestic interview, and neurological and primary cognitive examination for excluding history or presence of neurological disorders. The inclusion criteria were: i) age ≥ 18 years; ii) normal or corrected-to-normal vision excluding contact lenses; iii) a total score > 22.23 at the Montreal Cognitive Assessment, corresponding to the highest possible equivalent score interval (Santangelo *et al.* 2015); iv) written informed consent. Exclusion criteria were: i) presence of ophthalmic disorders or impairments in eyelid motility on clinical evaluation; ii) consumption of drugs acting on the central nervous system in the previous 24 h; iii) previous history of neurologic or psychiatric diseases.

### Procedure

After screening for inclusion/exclusion criteria, participants’ demographic and patients’ anamnestic information was collected. The level of consciousness and cognitive functioning were classified according to their best score on the repeated (at least five in two consecutive weeks) Coma Recovery Scale-Revised (CRS-R; Giacino *et al.* 2004) and on Levels of Cognitive Functioning (LCF; Hagen *et al.* 1979), respectively. Within 2 weeks from enrolment, each participant underwent EOG recording session, whilst seated in front of a plain wall, awake (i.e. with eyes open), in a quiet and dimly lit environment. Both healthy participants and patients were encouraged to stay relaxed and were never informed of the specific aim of blink recording to avoid any bias. Through each session, the examiner remained positioned beside the participant, but outside their visual field.

**Figure 1:**
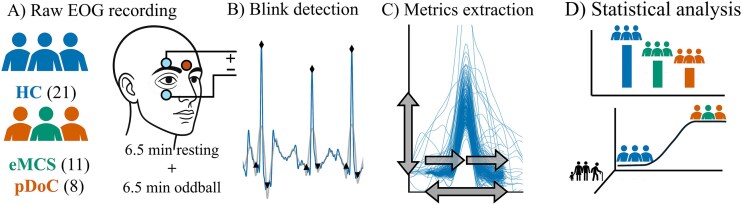
Overview of methods. (A) Raw EOG recording under resting state and auditory oddball stimulation; (B) blink detection and definition of onset and offsets, after Savitsky-Golay filtering in grey; (C) extraction of mean values of amplitude, duration, rise time, fall time; (D) statistical testing for group main effect and condition main effect, followed by demographics-adjusted testing with logistic regression model. Panels b and c make use of data from a real subject picked at random from HC group in resting state.

Vertical EOG was recorded at 1024 Hz by means of a pair of electrodes placed over the supraorbital ridge and below the infraorbital ridge of one eye, referenced to the nasion (Fig. 1A). Impedance was kept below 5 kΩ. Sessions took place between 10:00 AM and 5:00 PM: this time window is minimally influenced by circadian peaks of arousal in patients with pDoC (Wislowska *et al.* 2017) and is less influenced by fluctuations of EBR in healthy individuals (Barbato *et al.* 2000). Each session consisted of a rest condition (duration: 6.5 min) and an active auditory oddball task (duration: 6.5 min), with randomly intermixed tones (non-target: 500 Hz, overall probability: 80%, n = 312; target: 1000 Hz, overall probability: 20%, n = 78) presented with a 1-s inter-stimulus interval through headphones. If an EOG recording presented a considerable number of movement artefacts (i.e. ˃50% of recording), the acquisition was repeated the next available day. If the patients exhibited insufficient arousal, the CRS-R arousal facilitation protocol (Giacino *et al.* 2004) was administered. If the patient continued to keep their eyes closed, the recording session was rescheduled for the next available day.

In the cohort of patients, CRS-R and LCF scores were also gathered on the day of the EOG and considered for statistical analysis.

The investigators in charge of data collection also took care of the data entry. Patients’ data were collected and entered in a pseudonymized form into a password-protected, electronic database on REDCap (Research Electronic Data Capture), following as much as possible a forced-choice format. REDCap complies with the Health Insurance Portability and Accountability Act in compliance with security measures and therefore only contains data that has already been pseudonymized. The personal data of each patient were de-identified and replaced by an alphanumeric ID code in an ‘association key’ document accessible exclusively to the enrolling centre in a password-protected Excel file and eventually uploaded to REDCap.

### EOG blink waveform processing

All the processing was carried out with custom Python code available on GitHub (https://github.com/Leonardo-Corsi/blink-waveforms-sabi). The vertical EOG was resampled to 128 Hz and movements annotations were used to reject the 10 s preceding the annotation replacing the data with piecewise cubic Hermite interpolation. The EOG signal was then band-pass filtered between 1 Hz and 10 Hz with a zero-phase FIR filter.

Eye blinks were detected using the MNE analysis pipeline for EOG as the positive peaks exceeding an amplitude threshold set to 97.5th percentile of the filtered signal (Fig. 1B). The onset and offset of the blink waveform were defined as the lowest local minimum on each side of the peak within 500 ms, as found in a copy of the EOG signal filtered with a second order Savitzky–Golay filter with a length of 500 ms (Fig. 1B). Waveforms with total duration shorter than 100 ms were rejected. Finally, we rejected blink waveforms that were dissimilar from the other waveforms within the same subject, using the onset of the elbow in the median similarity cumulative distribution, as defined through 3-points linear dynamic programming (Truong *et al.* 2020). A baseline correction of each waveform was performed considering the first 50 ms after the onset. For each subject, the following measures were extracted: i) EBR, as the number of blinks divided by the total time minus intervals removed because of movements artefacts; ii) the variability of blink timing, estimated as the logarithm of the standard deviation of the inverse of blink-blink intervals that did not overlap with movement artefacts (log-inverse blink interval variability, LIBIV); iii) the amplitude at the blink peak, expressed in μV (Fig. 1C); iv) the total duration, expressed in ms (Fig. 1C); v) rise time and fall time, expressed in ms (Fig. 1C). The above-mentioned features were extracted from the 6.5-min recordings, excluding the first 30 s to minimize start-up transients.

### Statistical analysis

Descriptive statistics were expressed in terms of median (inter-quartile range, IQR) for continuous variables and as frequencies for categorical variables.

Demographic data (age, sex) were compared between healthy controls (HC) and sABI patient sample, and exploratively between HC, eMCS, and pDoC patients by means of the Kruskal-Wallis H test, the Mann–Whitney U test or the χ^2^-test, as appropriate. Moreover, anamnestic (aetiology, TPI) and clinical data (CRS-R total score, LCF score) were compared between eMCS and pDoC patients by means of the Mann–Whitney U test or χ^2^-test, as appropriate.

Afterwards, spontaneous eye blinking features (EBR, LIBIV, amplitude, duration, rise time, fall time) were compared across HC, eMCS, and pDoC patients using the Kruskal-Wallis H test followed, when significant (*P* < .05), by Conover-Iman post-hoc test at significance level of 0.05 for features with Holm-Bonferroni correction (Fig. 1D, top). Within-subject condition (rest, oddball) effects were assessed *via* the Wilcoxon signed-rank test.

To directly contextualize the observed variability, we computed the coefficient of variation (CV) in our dataset for the blink features with values reported in the literature for EBR in both healthy and pDoC patients and for blink duration in healthy subjects (Kleifges *et al.* 2017; Magliacano *et al.* 2021). Age and sex are also known to influence spontaneous blinking (Schellini *et al.* 2005; DeAngelis *et al.* 2019). Hence, we fitted binary logistic regression models for each blink feature and for each condition (resting, oddball) to evaluate differences between HC and sABI considering demographics (Fig. 1D, bottom). The dependent variable was group membership (0 = healthy, 1 = sABI); the single blink feature, age and sex were the predictor. More in detail, multivariate logistic regression models probability (Eq. 2), or alternatively the logarithmic odds (Eq. 2), of membership to the patient class:


(1) \begin{eqnarray*} P\left({sABI}_i=1\right)=\frac{1}{1+{e}^{-\left({\beta}_0+{\beta}_f{X}_{i,f}+{\beta}_{age}{X}_{i, age}+{\beta}_{sex}{X}_{i, sex}\right)}} \end{eqnarray*}

(2) \begin{eqnarray*} \mathit{\log}\left(\frac{P\left({sABI}_i=1\right)}{1-P\left( sAB{I}_i=1\right)}\right)={\beta}_0+{\beta}_f{X}_{i,f}+{\beta}_{age}{X}_{i, age}+{\beta}_{sex}{X}_{i, sex} \end{eqnarray*}

where $P\left({sABI}_i=1\right)$ is the probability that subject $i$ belongs to the sABI group; ${X}_{i,f}$, ${X}_{i, age},{X}_{i, sex}$ are the blink metric under test, age in years, sex as binary indicator (0 = female, 1 = male) for the subject $i$,all z-score standardized before regression to allow comparison of effect magnitudes across metrics. The model parameters are estimated by maximum likelihood estimation. For each coefficient, the Wald statistic for each blink metric is computed as ${z}_f={\hat{\beta}}_f/ SE\left({\hat{\beta}}_f\right)$ where $SE\left({\hat{\beta}}_f\right)$ is the standard error of the regression coefficient and we report the corresponding two-sided p-value is obtained from the standard normal distribution. To ease interpretation, we also report the odds ratios (ORs) obtained by exponentiation of the coefficient and their 95% confidence interval (CI):


(3) \begin{eqnarray*} O{R}_f&=\mathit{\exp}\left({\hat{\beta}}_f\right),\nonumber\\ C{I}_f&=\left[\mathit{\exp}\left({\hat{\beta}}_f-1.96\cdotp SE\left({\hat{\beta}}_f\right)\right),\kern0.5em \mathit{\exp}\left({\hat{\beta}}_f+1.96\cdotp SE\left({\hat{\beta}}_f\right)\right)\ \right] \end{eqnarray*}

ORs > 1 indicate higher odds of being a sABI group membership as the feature increases.

## Results

### Sample characteristics

A clinical sample of 19 patients with sABI, including eight patients with pDoC (5 in UWS, 3 in MCS) and 11 patients in eMCS was enrolled. In parallel, a sample of 21 HC was enrolled as a benchmark population. Descriptive statistics of demographics, anamnestic, and clinical information of the included participants are reported in Table 1. Patients with sABI were significantly older than HC. Within the sABI group, patients with pDoC had lower CRS-R total scores and lower LCF scores as expected from the diagnostic stratification. Characteristics at single-patient level are reported in Table 2.

**Table 1:** Descriptive statistics of the included participants as a function of the group (HC, n = 21; eMCS, n = 11); (pDoC, n = 8), expressed in terms of median (inter-quartile range, IQR) for continuous variables and as frequencies for categorical variables. Differences in age are reported for Mann–Whitney U-test as post-hoc after Kruskal-Wallis omnibus test. Differences in sex and aetiology were tested with χ^2^-test. Differences in TPI, CRS-R and LCF score between eMCS and pDoC were tested with Mann–Whitney U test. Significant differences in bold.

	HC	eMCS	pDoC	Statistical testing
Sex, M/F	13/8	5/6	4/4	χ^2^ = 0.890, *P* = .641
Age, years	30 (20)	62 (16)	55.5 (16.75)	U_HC,eMCS_ = 38.50, ***P***_**HC,eMCS**_ **= .002** U_HC,pDoC_ = 28.00, ***P***_**HC,pDoC**_ **= .007** U_pDoC,eMCS_ = 47.50, *P*_pDoC,eMCS_ = .804
Aetiology, TBI/non-TBI		2/9	2/6	χ^2^ = 0.130, *P* = .719
TPI, months		4.57 (5.47)	4.32 (6.51)	U_pDoC,eMCS_ = 42.0, *P*_pDoC,eMCS_ = .904
CRS-R, total score		23 (2)	6.5 (4)	U_pDoC,eMCS_ = 0.0, ***P***_**pDoC,eMCS**_ **< .001**
LCF		6 (2.5)	2 (1)	U_pDoC,eMCS_ = 1.5, ***P***_**pDoC,eMCS**_ **< .001**

Abbreviations: CRS-R, coma recovery scale-revised; eMCS, emerged from minimally conscious state; MCS, minimally conscious state; UWS, unresponsive wakefulness syndrome; LCF, level of cognitive functioning; F, female; M, male; TBI, traumatic brain injury; TPI, time-post-injury.

**Table 2:** Characteristics of the patients. CRS-R total score and relative sub-scores (auditory–visual-motor-oromotor/verbal-communication-arousal) are reported.

Patient	Sex	Age, years	Diagnosis	Aetiology	TPI, months	CRS-R	LCF
1	F	40–49	eMCS	Traumatic	4.57	23 (4-5-6-3-2-3)	7
2	F	20–29	MCS	Traumatic	15.47	9 (1-3-2-1-0-2)	3
3	M	60–69	eMCS	Vascular	4.47	23 (4-5-6-3-2-3)	6
4	F	50–59	MCS	Traumatic	2.37	11 (1-3-5-1-0-1)	3
5	M	70–79	eMCS	Anoxic	3.50	23 (4-5-6-3-2-3)	8
6	M	60–69	UWS	Vascular	4.60	5 (1-0-2-0-0-2)	2
7	M	60–69	eMCS	Vascular	16.03	20 (4-5-6-1-2-2)	5
8	F	50–59	eMCS	Neoplastic	9.43	23 (4-5-6-3-2-3)	8
9	M	80–89	UWS	Vascular	3.97	5 (1-0-2-1-0-1)	2
10	F	60–69	eMCS	Vascular	8.90	22 (4-5-6-3-2-2)	5
11	M	70–79	eMCS	Vascular	15.27	18 (2-4-6-3-1-2)	5
12	M	60–69	UWS	Neoplastic	9.70	6 (1-0-2-1-0-2)	2
13	F	50–59	eMCS	Vascular	7.17	20 (4-5-6-1-2-2)	3
14	F	60–69	eMCS	Anoxic	3.90	23 (4-5-6-3-2-3)	8
15	M	30–39	eMCS	Infectious	1.60	23 (4-5-6-3-2-3)	7
16	F	50–59	UWS	Anoxic	2.47	4 (1-0-0-1-0-2)	2
17	M	50–59	UWS	Vascular	4.03	7 (1-1-2-1-0-2)	2
18	F	10–19	eMCS	Traumatic	2.27	23 (4-5-6-3-2-3)	5
19	F	50–59	MCS	Anoxic	11.30	9 (2-0-2-2-1-2)	3

Abbreviations: CRS-R, coma recovery scale-revised; eMCS, emerged from minimally conscious state; MCS, minimally conscious state; UWS, unresponsive wakefulness syndrome; LCF, level of cognitive functioning; F, female; M, male; TBI, traumatic brain injury; TPI, time-post-injury.

### Blink features

**Figure 2:**
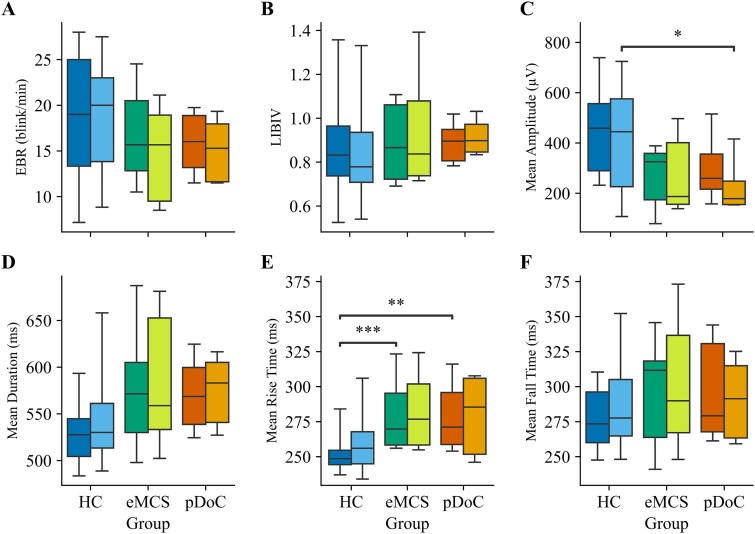
Boxplots of each feature in the three groups and in the two conditions (resting state in darker shade and oddball in brighter shade). Significance annotated according to Conover post-hoc test (^*^: *P* < .05, ^**^: *P* < .01), after Holm-Bonferroni correction. Abbreviations: eMCS, emerged from minimally conscious state; HC, healthy controls; LIBIV, logarithmic inverse blink interval variability; pDoC, prolonged disorder of consciousness.

The summary of each extracted blink feature is reported in Table 3 with their median and inter-quartile range (IQR) and shown graphically in Fig. 2.

**Table 3:** Median and IQR of each blink feature and condition for the three groups.

		HC	eMCS	pDoC
EBR	Resting	19.0 (13.3, 25.0)	15.7 (12.8, 20.5)	16.0 (13.2, 18.9)
	Oddball	20.0 (13.8, 23.0)	15.7 (9.5, 18.9)	15.3 (11.6, 18.0)
LIBIV	Resting	0.8 (0.7, 1.0)	0.9 (0.7, 1.1)	0.9 (0.8, 0.9)
	Oddball	0.8 (0.7, 0.9)	0.8 (0.7, 1.1)	0.9 (0.8, 1.0)
Amplitude	Resting	458.6 (289.7, 556.4)	325.3 (174.2, 358.5)	259.4 (216.3, 355.8)
	Oddball	444.7 (226.4, 575.6)	187.1 (156.1, 401.2)	178.6 (155.3, 248.2)
Duration	Resting	527.6 (504.4, 544.8)	571.5 (530.0, 605.0)	568.6 (538.7, 599.7)
	Oddball	530.1 (513.4, 561.1)	558.8 (533.3, 652.7)	583.1 (540.8, 605.0)
Rise Time	Resting	248.6 (244.4, 254.6)	269.8 (258.3, 295.3)	271.1 (258.7, 295.8)
	Oddball	256.0 (244.9, 267.8)	276.8 (258.4, 301.9)	285.4 (251.7, 306.0)
Fall Time	Resting	273.5 (260.0, 296.2)	311.7 (263.8, 318.3)	279.2 (267.8, 330.7)
	Oddball	277.7 (264.8, 305.1)	289.9 (267.2, 336.6)	291.4 (263.4, 314.8)

Abbreviations: EBR, eye blink rate; eMCS, emerged from minimally conscious state; LIBIV, logarithmic inverse blink interval variability; pDoC, prolonged disorder of consciousness.

Table 4 reports the Kruskal–Wallis (KW) main effects and Conover–Iman post-hoc tests. Post-hoc comparisons were computed for measures with KW *P* < .05: mean amplitude (resting, oddball), mean rise time (resting, oddball), and mean duration (resting). No statistically significant differences were observed between eMCS and pDoC for any blink feature in either condition. No group effect was found for EBR, LIBIV and fall time in both conditions and for mean duration during oddball. The Conover-Iman post-hoc test revealed statistically significant differences in amplitude and rise time between both eMCS and pDoC patients compared to HC, but not between eMCS and pDoC. After Holm-Bonferroni correction, mean rise time during the rest condition was found to be significantly longer in sABI than in HC (HC vs. eMCS: *P* = .001; HC vs. pDoC: *P* = .003). In the oddball condition, the group effect was not significant after correction for neither patient group. The mean duration only resulted in a non-significant trend for both patient cohorts when compared to HC. The group main effect for mean blink amplitude, after correction, remained significant only for HC compared to pDoC during oddball, leaving only a non-significant trend for eMCS patients compared to HC in both conditions. Within-group comparisons (resting versus oddball; Wilcoxon signed-rank) showed a condition effect only for mean amplitude in pDoC (W = 1, *P* = .020). No significant main effect of condition was found for the other features.

**Table 4:** Statistical testing of difference between groups for each feature in the two conditions (^*^:*P* < .05; ^**^:*P* < .01,^***^:*P* < .001). Adjusted *P*-values (Holm-Bonferroni) in brackets.

		Kruskal-Wallis			Conover-Iman		
		H (df = 2)	*P*	η^2^	HC vs. eMCS	eMCS vs. pDoC	HC vs. pDoC
EBR	Resting	1.4	.496	0.04			
	Oddball	4.35	.114	0.11			
LIBIV	Resting	0.34	.843	0.01			
	Oddball	3.01	.222	0.08			
Mean Amplitude	Resting	6.71	.035^*^	0.17	0.020^*^ (0.061)	0.892 (0.892)	0.051 (0.101)
	Oddball	6.92	.031^*^	0.18	0.053 (0.106)	0.520 (0.520)	0.016^*^ (0.049^*^)
Mean Duration	Resting	6.48	.039^*^	0.17	0.038^*^ (0.095)	0.780 (0.780)	0.031^*^ (0.093)
	Oddball	3.46	.177	0.09			
Mean Rise Time	Resting	14.72	.001^***^	0.38	*P* < .001 (0.001^***^)	0.823 (0.823)	0.001^**^ (0.003^**^)
	Oddball	6.17	.046^*^	0.16	0.025^*^ (0.075)	0.850 (0.850)	0.068 (0.136)
Mean Fall Time	Resting	2.1	.350	0.05			
	Oddball	0.63	.730	0.02			

Abbreviations: EBR, eye blink rate; HC, healthy controls; LIBIV, logarithmic inverse blink interval variability; eMCS, emerged from minimally conscious state; pDoC, prolonged disorder of consciousness.

CV for mean resting-state EBR was 38.3% in HC, 35.2% in eMCS, and 25.2% in pDoC. CV for mean duration was 9.4% in HC, 11.5% in eMCS, 7.4% in pDoC.

Table 5 summarizes the ORs and Wald tests of the logistic regression models when accounting for age and sex. The association of amplitude with group lost significance in both conditions, suggesting it is largely driven by demographics. In contrast, temporal metrics were significant discriminators after adjustment, in particular resting mean duration (*P* = .025) and resting mean rise time (*P* = .033) whilst resting mean fall time only approached the threshold of significance (*P* = .054). The EBR and LIBIV were not significant after adjustment.

**Table 5:** Logistic regression model fit for discrimination of HC and patients, fitted univariately with each feature and jointly with age and sex. All predictors are z-score standardized before regression. Odds ratios for the feature refer to the odds of participants being a sABI patient. *P*-values refer to Wald test (^*^:*P* < .05).

		OR (95% CI)	*P*
EBR	Resting	0.538 (0.237, 1.222)	.138
	Oddball	0.530 (0.226, 1.244)	.145
LIBIV	Resting	1.088 (0.483, 2.451)	.838
	Oddball	1.780 (0.761, 4.164)	.184
Amplitude	Resting	0.669 (0.266, 1.682)	.392
	Oddball	0.985 (0.342, 2.839)	.978
Duration	Resting	2.894 (1.145, 7.316)	.025^*^
	Oddball	1.960 (0.836, 4.596)	.122
Rise Time	Resting	2.521 (1.078, 5.895)	.033^*^
	Oddball	2.122 (0.904, 4.98)	.084
Fall Time	Resting	2.576 (0.986, 6.734)	.054
	Oddball	1.503 (0.626, 3.608)	.362

Abbreviations: EBR, eye blink rate; HC, healthy controls; LIBIV, logarithmic inverse blink interval variability; OR, odds ratio; sABI, severe acquired brain injury.

## Discussion

In this study we aimed at determining whether spontaneous eye blink features extracted from a short vertical EOG recording could provide objective and demographically robust electrophysiological indicators of cognitive alterations in patients with sABI. Building on previous evidence linking blinking dynamics to dopaminergic activity and attentional control, we analyzed both rate- and waveform-based blink features (EBR, amplitude, rise time, fall time, duration, and blink-blink interval variability) during resting state and an auditory oddball task. We sought to identify blink metrics that reliably differentiate sABI patients from healthy controls and that could potentially serve as bedside markers of central slowing associated with brain injury, supporting quantitative approaches in rehabilitation monitoring.

Our results show that temporal blink features, specifically rise time and duration, reflect reliable indicators of central drive slowing in patients with sABI, independently from demographics.

In contrast, blink amplitude appears highly age-sensitive and less suitable as a robust physiological marker in this population.

Whilst EBR has previously been shown to differentiate between *VS*/UWS and MCS (Magliacano *et al.* 2021) within a pDoC cohort. No differences were observed between pDoC and eMCS in the present study, indicating that blink features analyzed here were not associated with emergence from pDoC. We therefore moved beyond frequency-based measures by characterizing blink waveform dynamics, capturing a signature of central slowing intrinsic to sABI.

In univariate analysis, we found statistically significant differences between sABI patients and healthy controls for amplitude, duration and rise time, where instead fall time, EBR and LIBIV did not show statistically significant differences. In sABI patients, rise time and total duration were longer than in healthy controls, especially during resting state. After adjusting for age and sex, amplitude differences disappeared, confirming that peripheral biomechanical factors (e.g. eyelid tone or blink completeness) are strongly influenced by ageing (Sun *et al.* 1997; Talens-Estarelles *et al.* 2022). In contrast, only a subset of waveform features, specifically rise time and total duration, remained associated with the sABI group after adjustment, whereas amplitude did not, supporting a feature-specific interpretation of altered central control. These findings highlight the importance of moving beyond blink occurrence and interval-based measures towards a more detailed characterization of blink waveform dynamics.

Importantly, our findings are further supported by the relatively low variability observed for waveform-based temporal measures. Kleifges *et al.* (2017) reported resting-state EBR variability in HC corresponding to CV values in the range of ~40%–60%, depending on the dataset. Using mean and standard errors reported in Magliacano *et al.* (2021), it is possible to derive approximate resting-state EBR CV values of 90.6% and 121.7% in *VS*/UWS and 48.8% and 70.5% in MCS across two sessions. Here, we found that CV was broadly consistent with previous literature (i.e. 38.3% in HC, 35.2% in eMCS, and 25.2% in pDoC), confirming the intrinsic inter-individual variability of occurrence-based blink measures.

Blink duration showed relatively lower variability across groups (9.4% in HC, 11.5% in eMCS, 7.4% in pDoC whereas values of 25%–35% have been reported for healthy subjects by Kleifges *et al.* 2017), suggesting that waveform features capture more stable within-blink motor dynamics than blink frequency. The robustness and persistence of rise time and total duration after demographic adjustment therefore supports their interpretation as relatively robust, feature-specific indices of altered blink dynamics in sABI, whilst still warranting a preliminary and hypothesis-generating framing rather than a diagnostic one.

Interestingly, the observed effect of condition was that group differences were strongest at rest and attenuated during the auditory oddball task. This suggests that blinks elicited in externally cued contexts may partially override endogenous kinetic differences, whilst resting-state blinks better capture spontaneous dopaminergic and attentional drive. In this view, the resting condition provides a purer window into the endogenous motor and cognitive state, unconfounded by task-related modulation, which is particularly relevant in rehabilitation settings where task engagement is often limited.

### A joint mesocircuit interpretation for EBR and duration metrics

From a physiological point of view, an eye blink is a reciprocal pattern of transient inhibition of the levator palpebrae superioris and near-synchronous activation of orbicularis oculi motoneurons (Evinger *et al.* 1991). The basal ganglia-fronto-striatal network and premotor brainstem nuclei control blink occurrence and the time course of blink dynamics under tonic dopaminergic control (Jongkees and Colzato 2016; Maffei and Angrilli 2018; Bologna *et al.* 2024). Consistently with this view, patients with impaired dopaminergic transmission may show slowing and abnormalities of this pattern, as in the case of Parkinson disease (Cao *et al.* 2024). In patients with sABI and pDoC, the alteration in the anterior forebrain mesocircuit (including basal ganglia-fronto-striatal network) has been proposed to underline motor inertia and cortical reactivity (Schiff 2010). According to this model, widespread deafferentation leads to reduced thalamo-cortical drive and decreased striatal dopaminergic tone. The same dopaminergic circuits are involved in blink dynamics; therefore, the expected effects may include lower EBR and delayed triggering of levator palpebrae inhibition and orbicularis oculi contraction. This is consistent with previous results on EBR (Magliacano *et al.* 2021) as well as with increased duration-related blink metrics, observed in the present study.

Notably, amplitude lost significance after demographic correction, in line with the notion that amplitude is more strongly influenced by neuromuscular efficiency and eyelid biomechanics rather than by central neural drive. The combined prolongation of rise and fall times, leading to an extended overall blink duration, thus emerges as a potential marker of mesocircuit hypoactivity. From this perspective, blink waveform metrics may serve as non-invasive, indirect probes of dopaminergic function, complementing established electrophysiological or imaging approaches for assessing subcortical integrity.

### Limitations and future directions

The relatively small group size did not allow additional analyses, including comparisons within the pDoC subgroup and between traumatic and non-traumatic aetiologies, and limits the interpretability of the present findings. Although the logistic regression models yielded significant odds ratios for selected features, these results should be confirmed in a larger multicentre cohort and interpreted at the group level rather than as individual diagnostic markers.

Although the limited sample size prevented formal modelling of correlations between blink dynamics and clinical scores (CRS-R, LCF), this study opens a pathway towards using EOG-derived metrics as electrophysiological correlates of generalized slowing and impaired cognitive processes in sABI. Future research should examine whether blink timing parameters track behavioural signs of consciousness recovery, potentially serving as physiological markers of emerging command-following or attentional responsiveness that are crucial for rehabilitation planning. Such correlations could establish blink waveform metrics as adjunctive tools in clinical monitoring of patients with disorders of consciousness.

Furthermore, EOG recordings were obtained on a single occasion, precluding assessment of day-to-day or circadian stability and therefore the clinical minimum detectable change of each blink metric. Establishing intra-individual stability will be critical to determine whether these features can serve as reliable longitudinal biomarkers rather than cross-sectional indicators.

A further direction concerns multimodal integration. Since blink-related EEG components are associated with cortical arousal and executive control, combining EOG-derived features with EEG spectral and event-related measures may provide a more comprehensive characterization of the interaction between subcortical motor dynamics and cortical arousal processes. This multimodal perspective could help disentangle brainstem-level motor dynamics from cortical top–down influences, yielding interpretable and physiologically grounded indices of arousal-related and cognitive-motor processes along the rehabilitation course.

Finally, from a translational standpoint, the use of automated pipelines for blink detection, as implemented in this study, makes bedside assessment highly feasible. Short, resting-state EOG recordings could be integrated into continuous monitoring systems, enabling the development of low-cost, scalable *digital biomarkers* for neurorehabilitation settings.

## Conclusions

Our results suggest that specific blink waveform features, particularly duration and rise time, show consistent alterations in patients with sABI, reflecting a slowing of blink dynamics. Whereas low blink amplitude should be interpreted with caution, especially in older patients, prolonged blink duration and rise time may provide a more direct insight into central neural dynamics and are consistent with central slowing and altered dopaminergic and cortico-subcortical interactions.

Based on these findings, the present study suggests that a brief EOG recording can yield relevant metrics from blink waveform and timing that are independent from age and sex. The measurement is bedside feasible in clinical settings and is neither expensive nor time-consuming. Most importantly, this assessment requires no command-following from the patient. Indeed, these results contribute to the broader effort to identify physiological markers of altered motor and arousal-related processes in conditions where behavioural assessment is limited (Bodien *et al.* 2024). Because blinking requires minimal voluntary control, temporal slowing of this behaviour may reflect changes in the regulation of an otherwise automatic motor pattern, providing complementary physiological information in sABI. At this stage, though, these measures should be interpreted as group-level physiological markers rather than stand-alone diagnostic tools at the individual level. Future analysis integrating blink dynamics with quantitative EEG evaluation could further complement the behavioural assessment, as they can add objective markers of residual brainstem and corticobasal integrity aiding considerations within rehabilitation pathways for pDoC. Such approach could support longitudinal monitoring and contribute to the characterization of recovery trajectories in patients with sABI.

## Supplementary Material

OPEN_SCIENCE_BADGE_APPLICATION_FORM_niag043_updated

## Data Availability

The full data supporting this study are available with the code reproducing the article results at https://github.com/Leonardo-Corsi/blink-waveforms-sabi.
