# Supplementary material for: Demographics-robust spontaneous eye blinking slowing in patients with severe acquired brain injury
Source: Neurosci Conscious. 2026 Aug 12;2026(1):niag043. doi: 10.1093/nc/niag043 (PMC13464740; doi:10.1093/nc/niag043)
Supplement: Supplementary file 1 [file open_science_badge_application_form_niag043_updated.docx]

v **OPEN SCIENCE BADGE APPLICATION FORM**

**Open Data Badge**

Please provide the URL, DOI, or other permanent path for accessing the data in a public, open access repository.

I

<https://github.com/Leonardo-Corsi/blink-waveforms-sabi/releases/tag/v1.1.0>

Is there sufficient information for an independent researcher to reproduce the reported results? If no, explain.

Yes

**Open Materials Badge**

Please provide the URL, DOI, or other permanent path for accessing the materials in a public, open access repository.

I

<https://github.com/Leonardo-Corsi/blink-waveforms-sabi/releases/tag/v1.1.0>

Is there sufficient information for an independent researcher to reproduce the reported methodology? If no, explain.

Yes

**Preregistered Badge**

Please provide the URL, DOI, or other permanent path to the registration (and, if applicable, the analysis plan) in a public, open access repository.

I

https://clinicaltrials.gov/study/NCT06464549

Was the plan preregistered prior to the examination of the data or observing the outcomes? If no, explain.

Yes

Were there additional registrations for the study other than the one reported? If yes, provide links and explain.

No

For Preregistered and Analysis plan badge: were there any changes to the preregistered analysis plan for the primary confirmatory analysis? If yes, explain.

No preregistered strict analysis plan

For Preregistered and Analysis plan badge: are all of the analyses described in the registered plan reported in the article? If no, explain.

No preregistered strict analysis plan
